# UV-Deprived Coloration Reduces Success in Mate Acquisition in Male Sand Lizards (*Lacerta agilis*)

**DOI:** 10.1371/journal.pone.0019360

**Published:** 2011-05-13

**Authors:** Mats Olsson, Staffan Andersson, Erik Wapstra

**Affiliations:** 1 School of Biological Sciences, University of Sydney, Sydney, Australia; 2 Department of Zoology, University of Gothenburg, Gothenburg, Sweden; 3 School of Zoology, University of Tasmania, Hobart, Tasmania, Australia; University of Jyväskylä, Finland

## Abstract

**Background:**

Recent work on animal signals has revealed a wide occurrence of UV signals in tetrapods, in particular birds, but also in lizards (and perhaps other Squamate reptiles). Our previous work on the Swedish sand lizard (*Lacerta agilis*) has verified, both in correlative selection analyses in the wild and with laboratory and field experiments, the importance of the green ‘badge’ on the body sides of adult males for securing mating opportunities, probably mostly through deterring rival males rather than attracting females. The role of UV in communication has, however, never been examined.

**Methodology/Principal Findings:**

Here we show that when measured immediately after spring skin shedding, there is also signaling in the UV. By UV-depriving the signal (reflectance) with sun block chemicals fixated with permeable, harmless spray dressing, we show that males in the control group (spray dressing only) had significantly higher success in mate acquisition than UV-deprived males.

**Conclusions/Significance:**

These results suggest that at least two colour traits in sand lizards, badge area and UV, contribute to rival deterrence and/or female choice on UV characters, which elevates success in mate acquisition in UV intact male sand lizards.

## Introduction

The study of animal communication is a complex science addressing a wide range of multi-layered questions, such as how a signal is emitted (e.g., visually, acoustically etc), how it is perceived (e.g. the spectral range and sensitivity of colour vision), and of course the adaptive reasons for signaling (e.g., deterring rivals, attracting mates). Any and all of these factors interact to mold signal selection in the wild, and mediate the type and degree of honest indication of some aspect of sender ‘quality’, which is expected in any evolutionarily stable signal trait [1]–[4].

Early work suggested that ‘badges of status’ would be beneficial to both signalers and receivers, since they would cut costs of contests to both contestants if an outcome would be predictable [5]. Subsequent work has debated whether merely ‘social costs’, in the absence of developmental costs, really are sufficient to guarantee honest and evolutionarily stable signaling, conveying aspects of ‘quality’, such as fighting or parental ability [6], [7]. Nevertheless, there is no lack of examples of seemingly ‘cheap’ yet adaptive badges in the recent literature, such as wing epaulettes in birds [8] and badges of bright colours in lizards [9].

The bright green colour ‘badge’ of our model species, the Swedish sand lizard (*Lacerta agilis*), shows spectral reflectance peaks in both green (ca 540 nm) and the UV (ca 340 nm). However, when we first investigated these traits in the mid 90's, neither the UV-component nor other aspects of colour as such (spectral shape) were identified; in particular, we dismissed the UV effect based on data from males caught at spring emergence from hibernation (with the rationale that signals present at this time are the most likely to reflect early male resource holding power when core areas of male home ranges are contested [9]). However, this ignores that the exuvia may not show the same spectroradiometry characteristics as a newly shed animal at maximal brightness. Instead, our signaling work on sand lizards has since mostly focused on the link between badge size (proportion green colour on a male's body sides), signaling, and fitness parameters in field and laboratory studies [9]–[12]. This work showed, for example, that badges contribute to mate acquisition; in smaller than average males experimental increment of badge size increased mate acquisition by 400 percent [12].

**Figure 1:**
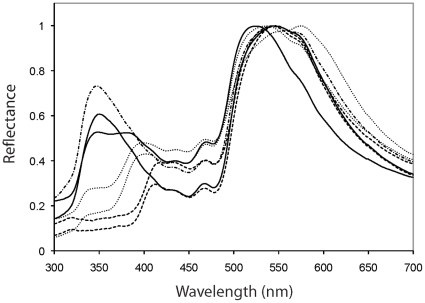
UV/VIS spectral reflectance from sand lizard flanks: wild (long-short dash), control-manipulated (solid lines), UV-manipulated 1 hour (dash), and 30 days (points) after treatment. Note the considerable UV-reduction also after 30 days in the wild. Spectra are set to equal brightness, in order to see spectral shape (i.e., colour) more precisely.

Given the renewed interest in UV signaling in both vertebrates and invertebrates (e.g., [13]–[21]), we revisited this research area in 2007. A pilot study confirmed that recently shed males indeed showed a much stronger reflectance peak in the UV spectrum (ca 340 nm, Fig. 1). This agrees with reflectance data from male *Lacerta agilis* in Pyrenees populations during the mating season [19]. We therefore designed an experiment in 2008 to test the proposition that UV blockage would interfere with rival and partner communication and compromise mate acquisition.

## Materials and Methods

The field work in this population (Asketunnan, Sweden ∼N57°22′ E11°58′) follows a well-established protocol that has been reported on in previous work (e.g., [9]–[12], [22]). In short, sand lizards (*Lacerta agilis*) are small (to 20 g), ground-dwelling lizards. Eighty five males and eighty females are individually marked short term by putting a uniquely numbered cloth tape on their backs. Males observed courting, copulating or mate guarding females were classified as partners. The sex ratio in the Asketunnan population is approximately 1∶1, the capture rate of adults is >90% and, thus, the observations made of the lizards in the current paper are based on nearly complete coverage of the adult population. That said, scored mating success of adult males is known to covary with the number of times males are observed, which was therefore controlled for in our analyses. All adults were weighed (to the nearest 0.1 g), measured (snout-vent and total length to the nearest 1 mm), and a 50 µl blood sample was taken from *vena angularis* (in the corner of the mouth) of both males and females and stored in 70% alcohol for later molecular genetic analysis. Males and females were then released at the place of capture and monitored during the mating season (ca seven weeks) every day that weather permitted.

Females were immediately released at the place of captured. Males were accumulated at daily field captures over a ten day period and stored at +8°C in a constant temperature room, awaiting a synchronized release of all males immediately after being weighed, measured, marked and treated with UV blocker (released 2 May 2008). Representative radiospectrometric analysis of UV blockage effects were performed at release and after three weeks (Fig. 1) to verify that our UV blockage had the desired long-term effects. The second measure after three weeks was virtually indistinguishable from the first (Fig. 1). The UV blockage was performed by gently rubbing +50 SPF (‘sun protection factor’; Vichy Laboratoires, Capital Soleil, Very High Protection] on every second male (*n* = 43, for controls, *n* = 42) in an Excel size-sorted data set of the captured males in storage. This ensured that UV-blocked and control males did not differ in snout-vent length, mass or body condition (*p*>0.14 for all three of these traits). Thereafter every male was sprayed with a vapour-permeable spray dressing, used to treat superficial human wounds (Smith & Nephew, Hull, England). Neither the storage at cool temperatures nor the spray dressing have any detrimental effects [12], and the UV block was developed for humans and appeared biologically inert on lizard skin (no apparent fading or discoloring was observed).

After the morphology data had been collected and the lizards treated, they were released at random places of capture (i.e., randomizing sites that were, at capture, potentially further or closer from females) and monitored for associations with partners (facilitated by the prolonged mate guarding, [22]) every day of the mating season when the weather permitted lizard activity (3 May–20 June, number of observation days, *n* = 26). Thus, our procedure also eliminates variation in male spring emergence (since all males are released simultaneously). Our work was approved by the Animal Ethics Committee, University of Gothenburg.

Our statistical analyses involved two approaches: (a) we first performed a homogeneity of slopes regression analysis with number of partners as response variable and treatment (UV-blocked vs. controls), number of observations of a male and its interaction with treatment, and male snout-vent length as covariate. However, because of some non-normality of the data (over-representation of zero pairing success), we (b) also performed an analysis more robust to deviation from normality using a logistic regression with an ordered cumulative logit model with the same trait variables.

## Results

**Figure 2:**
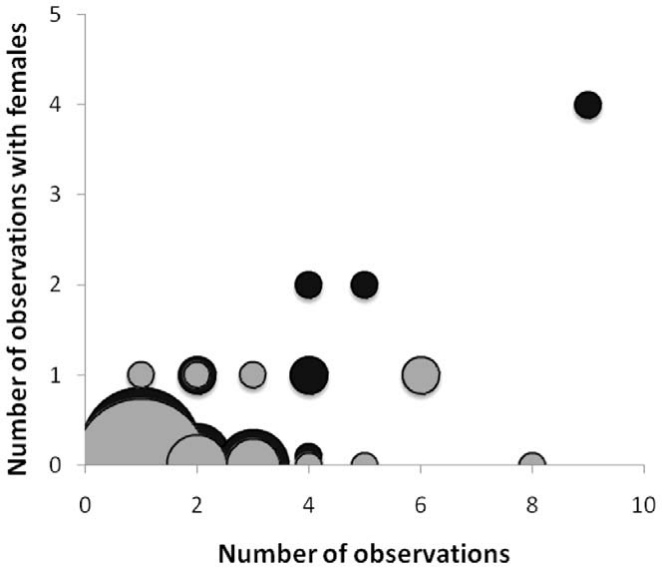
Mating success in male sand lizards depending on UV reduction (grey) versus control males (black). Increment symbol size represents increasing number of observations of males from 1 (smallest) to 24 (largest).

There was no difference in the mean number of observations of UV-reduced and control males (mean number of re-observations, 2.1±0.24, range 1 to 8, and 1.93±0.24, range 1 to 9, for control and UV-reduced males, respectively; T-test, *t* = 0.61, *P* = 0.54). Across treatment and control males, the number of observations of a male after release was correlated with the number of times he was seen courting a female (*r_s_* = 0.49, *P*<0.0001, *N* = 85). We therefore incorporated male number of re-sightings in our analysis of treatment effects on number of females paired. UV-blocked males had an average of 0.12 female pairing observations per male (±0.049, SE, *N* = 43), whereas the corresponding number for control males was three times as high (0.31±0.12, *N* = 42). The regression analysis was globally significant (*F*_3, 81_ = 40.7, *P*<0.0001, *R* ^2^ = 0.60), and had significant independent effects of treatment (*F* = 15.4, *P*<0.0002, d.f. = 1), number of observations (*F* = 82.9, *P*<0.0001, d.f. = 1), and their interaction (*F* = 39.7, *P*<0.0001; Fig. 2). Body size (SVL) was backwards eliminated from the final model (*P*>0.25). Our cumulative, ordered logistic regression largely agreed with these results (Global model Likelihood ratio *X* ^2^ = 35.0, *P*<0.0001, d.f. = 4). The number of observations of a male significantly affected the number of females he was observed with (Wald *X* ^2^ = 15.06, *P* = 0.0001), the treatment x observation interaction remained significant (Wald *X* ^2^ = 6.03, *P* = 0.014), while the treatment effect per se fell just short of significant (Wald *X* ^2^ = 3.07, *P* = 0.079; Fig. 2).

## Discussion

Our results show slight discrepancy between the logistic regression and the linear multiple regression analysis. However, we know from previous work [22] that the number of observations per male influences estimates of mate acquisition (number of females seen courting), and that this effect is modified by UV reduced signaling. Thus, it can be argued that the significant interaction term in both analyses is the correct unit of analysis, and it is significant in both cases. How robust are these results? The current study is specifically aimed at analyzing mate acquisition success in relation to UV blockage. Thus, analyzing access to females is a more appropriate level of analysis than tallying molecularly assigned offspring, since this level would include sperm competition and cryptic female choice effects [9], [23]. Regardless, male access to females is tightly correlated with probability of paternity for a given female [24] and hence our analysis should represent fitness consequences of UV-signaling, independent of badge signaling.

Our previous work [9] shows the effect of the area of nuptial coloration for successful mate acquisition and that males are likely to use both badge cues and other coloration for assessing rival fighting ability and to avoid repeating contests with other males [9]–[11]. How UV signaling adds additional information, or makes already described traits such as size and fighting ability more (or less) easily or accurately perceived cannot be deduced from the current experiment. However, since UV blocking has an effect on mate acquisition across the male size distribution when badge and other colour traits are unmanipulated, this seems to suggest that UV signaling is universally employed in all males and perhaps more important for conveying mere presence than fighting ability [9].

Our results and interpretations also agree with those of two previous studies on the role of UV in lizard communication. Stapley and Whiting [25] showed with a field experiment that males with reduced UV signals in *Platysaurus broadleyi* were more likely to be challenged by rivals, and Bajer et al. [26] showed that male green lizards (*Lacerta viridis*) with reduced UV signals were less spatially associated with by females. In sand lizards, we have never been able to demonstrate that there is female choice on male colour traits whereas there are strong effects of male green badges on male contest behavoiurs [9], [10]. Thus, we conclude that male UV reduction in this species compromises mate acquisition but that it is unresolved in free-ranging animals whether this is a combined effect of male-male rivalry and female choice on UV components of signalling.

In summary, our field experiment demonstrates a technique for long-term elimination of UV signaling in free-ranging lizards, which reduces success in mate acquisition, probably through reduced deterrence of rivals.

## References

[1] Andersson M (1986). Evolution of condition-dependent sex ornaments and mating preferences: sexual selection based on viability differences.. Evolution.

[2] Grafen A (1990a). Sexual selection unhandicapped by the Fisher process.. J Theor Biol.

[3] Grafen A (1990b). Biological signals as handicaps.. J Theo Biol.

[4] Hasson O (1997). Towards a general theory of biological signaling.. J Theor Biol.

[5] Rohwer S (1976). The evolution of reliable and unreliable badges of fighting ability.. Am Zool.

[6] Zahavi A (1977). The cost of honesty (Further remarks on the handicap principle).. J Theor Biol.

[7] Searcy WA, Nowicki S (2005). The evolution of animal signaling systems..

[8] Pryke S, Andersson S (2003). Carotenoid-based epaulettes reveal male competitive ability: experiments with resident and floater red-shouldered widowbirds.. Anim Behav.

[9] Olsson M (1994a). Nuptial coloration in the sand lizard (*Lacerta agilis*): an intrasexually selected cue to fighting ability.. Anim Behav.

[10] Olsson M (1994b). Rival recognition affects male contest behavior in sand lizards *Lacerta agilis*.. Behav Ecol Sociobiol.

[11] Olsson M (1994c). Why are sand lizard *Lacerta agilis* males not equally green?. Behav Ecol Sociobiol.

[12] Anderholm S, Olsson M, Wapstra E, Ryberg K (2004). Fit and fat from enlarged badges.. Biol Lett.

[13] Tovée MJ (1995). Ultra-violet photoreceptors in the animal kingdom: their distribution and function.. Trends Ecol Evol.

[14] Andersson S, Amundsen T (1997). Ultraviolet colour vision and ornamentation in blue throats.. Proc Roy Soc Lond B.

[15] Bennett ATD, Cuthill IC, Partridge JC, Lunau K (1997). Ultraviolet plumage colors predict mate preferences in starlings.. Proc Natl Acad Sci USA.

[16] Delhey K, Johnse A, Peters A, Andersson S, Kampenaers B (2003). Paternity analysis opposing selection on crown coloration in the blue tit (*Parus caeruleus*).. Proc Roy Soc Lond B.

[17] Losey GS (2003). Crypsis and communication functions of UV-visible coloration in two coral reef damselfish *Dascyllus aruanus* and *D reticulates*.. Anim Behav.

[18] Li D, Lim LM (2004). Ultraviolet cues affect the foraging behaviour of jumping spiders.. Anim Behav.

[19] De Lanuza GPI, Font E (2007). Ultraviolet reflectance of nuptial coloration in sand lizards (*Lacerta agilis*) from the Pyrenees.. Amphib Reptil.

[20] Martin J, Lopez P (2009). Multiple color signals may reveal multiple messages in male Schreiber's green lizard *Lacerta schreiberi*.. Behav Ecol Sociobiol.

[21] Whiting MJ, Stuart-Fox DM, O'Conner D, Firth D, Bennett NC (2006). Ultraviolet signals ultra-aggression in a lizard.. Anim Behav.

[22] Olsson M, Wapstra E, Madsen T, Silverin B (2000). Testosterone Ticks and Travels: a test of the immunocompetence-handicap hypothesis in free-ranging lizards.. Proc Roy Soc B.

[23] Olsson M, Shine R, Madsen T, Gullberg A, Tegelström H (1996). Sperm selection by females.. Nature.

[24] Gullberg A, Olsson M, Tegelström H (1997). Male mating success reproductive success and multiple paternity in a natural population of sand lizards: behavioural and molecular genetics data.. Mol Ecol.

[25] Stapley J, Whiting MJ (2006). Ultraviolet signals fighting ability in a lizard.. Biol Lett.

[26] Barja K, Molnár O, Török J, Herczeg G (2010). Female European green lizards (Lacerta viridis) prefer males with high ultraviolet throat reflectance.. Behav Ecol Sociobiol.

